# Transcriptome Analysis Reveals the Potential Key Genes in Nutritional Deposition in the Common Carp (*Cyprinus carpio*)

**DOI:** 10.3390/ani14131939

**Published:** 2024-06-30

**Authors:** Yunya Wu, Pengfei Xiao, Hang Sha, Xiangzhong Luo, Guiwei Zou, Hongwei Liang

**Affiliations:** 1 Yangtze River Fisheries Research Institute, Chinese Academy of Fisheries Science, Wuhan 430223, Chinash1812@yfi.ac.cn (H.S.);; 2 College of Fisheries and Life Science, Shanghai Ocean University, Shanghai 201306, China

**Keywords:** *Cyprinus carpio*, nutritional deposition, transcriptome, unsaturated fatty acids, amino acid

## Abstract

**Simple Summary:**

The common carp (*Cyprinus carpio*) is a significant aquaculture species in China. This study sought to identify the critical genes and signaling pathways that regulate muscle nutritional composition by analyzing muscle nutritional content and transcriptome data from liver and muscle tissues across three different carp varieties. The results revealed that among the three varieties, FFRC carp exhibited the highest levels of crude fat, total n-3 polyunsaturated fatty acids, and total n-6 polyunsaturated fatty acids in its muscle tissue. This was associated with the significant upregulation of genes in the liver involved in the activation and transportation of fatty acid as well as biosynthesis and elongation of long-chain unsaturated fatty acids. Additionally, Huanghe carp demonstrated the highest muscle crude protein and amino acid content. This was correlated with significant changes in the expression of genes related to amino acid metabolism. This research lays a theoretical groundwork for the genetic enhancement of carp, aimed at optimizing its nutritional composition.

**Abstract:**

The common carp (*Cyprinus carpio*) is one of the most important aquaculture species in China, known for its remarkable adaptability and nutritional profile. However, the specific molecular response mechanisms regulating the nutritional deposition of carp remain inadequately elucidated. This study conducted a comprehensive analysis of muscle nutritional content and transcriptome data from liver and muscle tissues of three distinct carp varieties. The aim was to elucidate the key genes and signaling pathways that regulate muscle nutritional composition in carp. The findings revealed that FFRC carp (FFRC) exhibited significantly higher levels of crude fat, total n-3 polyunsaturated fatty acids, and total n-6 polyunsaturated fatty acids in muscle tissue compared to Ying carp (YC) and Huanghe carp (HC) (*p* < 0.05). Transcriptomic analyses correlated these elevated levels with a marked upregulation of genes involved in the activation and transportation of fatty acid (*fabp7*, *acsl5*, *acsbg2*) as well as biosynthesis and elongation of long-chain unsaturated fatty acids (*elovl2*, *fads2*) within the liver. Furthermore, the flavor amino acid, essential amino acids, and crude protein content in the muscle of HC were significantly higher than in FFRC and YC (*p* < 0.05). Transcriptomic analyses indicated that this was associated with significant changes in the expression of genes related to amino acid metabolism (*asns*, *alt*, *ldha*, *glul*, *setd*, *prodh*, *l3hypdh*, *hoga1*) within their muscle tissue. This research provides a theoretical foundation for the precise modulation of the muscle nutritional composition in carp.

## 1. Introduction

The common carp (*Cyprinus carpio*) is extensively cultivated across various freshwater environments, particularly in Asia and Europe [1]. With a rich cultivation history spanning thousands of years, this species plays a pivotal role not only due to its substantial economic value but also because of its remarkable adaptability and nutritional profile [2]. Considering the escalating demand for health-conscious food options, recent scientific endeavors have increasingly focused on improving the nutritional value of the common carp [3].

Genetic enhancements have proven effective for augmenting phenotypic traits in the common carp. Through selective breeding, researchers have developed carp varieties with faster growth rates and greater adaptability [4]. Currently, there are over 25 recognized varieties of carp in China. Among them, the Ying carp (YC) variety was developed by the Yangtze River Fisheries Research Institute of the Chinese Academy of Fisheries Sciences, through hybridization between a female scattered mirror carp (♀) and fertile nucleocytoplasmic hybrids (♂, produced by transplanting the nuclei from the common carp, *Cyprinus carpio Red var vuyuanensis*, into enucleated eggs of the crucian carp). The Huanghe carp (HC) variety was selectively bred by the Henan Provincial Fisheries Science Research Institute, derived from wild strains of Huanghe carp. The FFRC carp (FFRC) variety was cultivated by the Freshwater Fisheries Research Center of the Chinese Academy of Fishery Sciences, using selective breeding techniques involving Jian carp and wild Huanghe carp [5].

The muscle tissue of the common carp, the species’ most valuable edible component, is highly nutritious and enriched with both essential and non-essential amino acids [6]. It contains high levels of essential amino acids such as lysine, methionine, leucine, and tryptophan, which are crucial for human growth, development, and health maintenance [7]. Although the fat content in carp muscle is relatively low, it is a rich source of healthy unsaturated fatty acids, particularly omega-3 fatty acids, including eicosapentaenoic acid (EPA) and docosahexaenoic acid (DHA) [8]. These fatty acids are particularly beneficial for heart health, can reduce the risk of cardiovascular diseases, and play a significant role in brain health and vision development [9]. The nutritional composition of carp muscle can be modified by adjusting feed components. Variations in protein sources can alter muscle amino acid composition [10], while increasing fish or vegetable oil in the diet can enhance polyunsaturated fatty acid (PUFA) concentration, particularly omega-3 acids [11,12]. Moreover, the mTOR signaling pathway and microRNAs may play crucial roles in the regulation of muscle protein deposition in fish [13,14,15]. Nevertheless, the specific molecular response mechanisms regulating the nutritional deposition of carp remain unclear.

The development of transcriptomic technology has provided unprecedented opportunities for understanding the intricacies of biological systems. In the field of aquatic sciences, transcriptomics has been widely applied to study fish growth, reproduction, disease resistance, and responses to environmental changes [16,17]. In this study, we examine three varieties of common carp, employing comprehensive transcriptome sequencing of liver and muscle tissues to investigate the principal genes and signaling pathways that regulate muscle nutritional deposition. This research aims to enhance our fundamental understanding of carp nutritional deposition and provide a scientific foundation for the genetic enhancement of common carp and other aquacultured fish species, thereby promoting the sustainable development of the aquaculture industry.

## 2. Materials and Methods

### 2.1. Experimental Fish and Sample Collection

An eight-month rearing trial (from April to November 2023) was conducted at Liangzi Lake Experimental Farm of the Yangtze River Fisheries Research Institute, Chinese Academy of Fisheries Sciences. One-year-old YC was provided by the Yaowan Experimental Farm of the Yangtze River Fisheries Research Institute. One-year-old HC was collected from the Zhongmou Carp Breeding Base of the Henan Provincial Fisheries Science Research Institute. One-year-old FFRC was collected from the Shangqiu Liangyuan District Wenfeng Aquaculture Cooperative. To maintain uniform aquaculture conditions for the three varieties of carp, a pond measuring 666.67 square meters was partitioned into three smaller ponds using fishing nets. Each small pond was stocked with 300 fish. The fish were fed on a commercial diet daily at 09:00 and 16:00.

At the end of the experiment, the fish were starved for 24 h and then anesthetized with 100 mg/L anesthetics (MS-222; Sigma, Shanghai, China). Fifty individuals were randomly selected and weighed from three varieties of carp (YC final weight: 867.84 ± 204.936 g, HC final weight: 876.76 ± 172.058 g, FFRC final weight: 801.34 ± 184.643 g). Three individuals per variety were randomly selected, and samples of muscle and liver tissues were collected. These samples were then rapidly frozen in liquid nitrogen and stored at −80 °C for subsequent analysis.

### 2.2. Muscle Chemical Composition Determination

#### 2.2.1. Proximate Composition Determination

According to the methods described by the Association of Official Analytical Chemists (AOAC), the crude protein (AOAC 981.10), crude lipid (AOAC 948.15), moisture (AOAC 950.46, 105 °C), and ash (AOAC 920.153) contents of the muscle were determined.

#### 2.2.2. Amino Acid Analysis

The frozen muscle tissue samples (25 ± 2 mg), spiked with 200 μL of cold methanol, were homogenized in a frozen state by a TissueLyser (JX-24; Jingxin, Shanghai, China) with beads at 40 Hz for 4 min. After centrifugation (15,000× *g* and 4 °C) for 15 min, the whole supernatant was evaporated to dryness under nitrogen gas, and the dried residues were derivatized at room temperature for 10 min after additions of 50 µL sodium carbonate and 50 µL benzoyl chloride (2%). The derivatized samples were isometrically mixed with a stable isotope labeled according to internal standards. The quality control (QC) sample was obtained by isometrically pooling all the prepared samples. A solution of multiple standards with 5 μM was prepared by mixing a single standard stock solution. The calibration curve solutions were obtained by serially diluting the mixed standard working solution and isometrically mixing with internal standards, respectively. The samples of the calibration curve covered a range of 0.01–5 μM. The samples were detected and analyzed by using liquid chromatography–tandem mass spectrometry (UHPLC–MS/MS) analysis, which was performed on an Agilent 1290 Infinity II UHPLC system (Agilent Technologies, Santa Clara, CA, USA) coupled to a 6470A Triple Quadrupole mass spectrometer (Agilent Technologies, Santa Clara, CA, USA).

#### 2.2.3. Fatty Acid Analysis

Approximately 25 mg of the sample was weighed, 100 µL of deionized water was added, and it was subjected to homogenization through freeze-crushing at 40 Hz for 4 min. The homogenate was transferred into a glass tube. To ensure complete transfer, the original container was rinsed with 100 µL of ultrapure water and combined with the homogenate in a glass tube. Total amounts of 100 µL of the internal standard (nonadecanoic acid), 500 µL of 10% acetyl chloride (dissolved in methanol), and 500 µL of n-hexane (containing butylated hydroxytoluene) were introduced to the mixture. The resultant mixture was incubated at 95 °C in a water bath for one hour, then cooled, centrifuged (10,000× *g*, 5 min), and the upper n-hexane phase collected for further analysis. For the preparation of quality control samples, an appropriate volume taken from all the samples was mixed and the same steps were used in the sample preparation method. For the quantitative standard curve, the sample was prepared by gradient dilution of the fatty acid methyl ester mixed standard stock solution, combined with methyl nonadecanoate. The samples were detected using gas chromatography–tandem mass spectrometry (GC–MS, 7890A; Agilent Technologies, Santa Clara, CA, USA) equipped with a Triple-Axis detector and a 30 m × 0.25 mm fused silica capillary column (inert XL EI/CI MSD, 5975C; Agilent Technologies, Santa Clara, CA, USA).

### 2.3. RNA Extraction, Library Construction, Sequencing, and Assembly

RNA extraction from liver and muscle tissues was performed using the TRIzol Reagent Kit (Thermo Fisher Scientific Inc., Waltham, MA, USA), following the manufacturer’s instructions. The degradation and contamination of RNA were determined by 1% agarose gel electrophoresis. The concentration and quality of RNA were determined using a NanoDrop spectrophotometer (Thermo Fisher Scientific Inc., Waltham, MA, USA). The integrity of RNA was evaluated using an Agilent 2100 Bioanalyzer (Agilent Technologies, Santa Clara, CA, USA). Three micrograms of RNA were used as input material for the RNA sample preparations. Sequencing libraries were generated according to the following steps. Firstly, mRNA was purified from total RNA using poly-T, oligo-attached, magnetic beads. Fragmentation was carried out using divalent cations under elevated temperature in an Illumina proprietary fragmentation buffer. First-strand cDNA was synthesized using random oligonucleotides and Super Script II. Second-strand cDNA synthesis was subsequently performed using DNA Polymerase I and RNase H. Remaining overhangs were converted into blunt ends via exonuclease/polymerase activities and the enzymes were removed. After adenylation of the 3′ ends of the DNA fragments, Illumina PE adapter oligonucleotides were ligated to prepare for hybridization. To select cDNA fragments of the preferred 400–500 bp in length, the library fragments were purified using the AMPure XP system (Beckman Coulter, Beverly, CA, USA). DNA fragments with ligated adaptor molecules on both ends were selectively enriched using an Illumina PCR Primer Cocktail in a 15-cycle PCR reaction. Products were purified (AMPure XP system) and quantified using the Agilent high-sensitivity DNA assay on a Bioanalyzer 2100 system (Agilent Technologies, Santa Clara, CA, USA). The sequencing library was then sequenced on Illumina NovaSeqTM 6000 (Illumina, San Diego, CA, USA)

### 2.4. Transcriptome Analysis

#### 2.4.1. Quality Control and Read Mapping

Samples were sequenced on the platform to obtain image files, which were transformed by the software of the sequencing platform, and the original data in FASTQ format (raw data) were generated. Sequencing data contained several connectors and low-quality reads, so we used fastp (0.22.0) software to filter the sequencing data to obtain high-quality sequences (clean data) for further analysis. The reference genome and gene annotation files were downloaded from a genome website. The filtered reads were mapped to the reference genome using HISAT2 (v2.1.0).

#### 2.4.2. Expression Analysis and Differential Expression Analysis

HTSeq (v0.9.1) statistics was used to compare the read count values on each gene as the original expression of the gene and then FPKM was used to standardize the expression. Then, the difference expression of genes was analyzed by DESeq (v1.38.3) with the following screened conditions: expression difference multiple |log2FoldChange| > 1, significant *p*-value < 0.05. At the same time, we used the R language Pheatmap (v1.0.12) software package to perform the bidirectional clustering analysis of all different genes of samples. We created a heatmap according to the expression level of the same gene in different samples and the expression patterns of different genes in the same sample with the Euclidean method, to calculate the distance, and the complete linkage method, to cluster.

#### 2.4.3. Enrichment Analysis

We mapped all the genes to terms in the Gene Ontology (GO) database and calculated the numbers of differentially enriched genes in each term. Using topGO (v2.50.0) to perform GO enrichment analysis on the differential genes (all DEGs/up DEGs/down DEGs), we calculated the *p*-value by the hypergeometric distribution method (the standard of significant enrichment was a *p*-value < 0.05) and found the GO term with significantly enriched differential genes to determine the main biological functions performed by differential genes. ClusterProfiler (v4.6.0) software was used to carry out the enrichment analysis of the Kyoto Encyclopedia of Genes and Genomes (KEGG) pathway of differential genes, focusing on the significant enrichment pathway with a *p*-value < 0.05. The Gene Set Enrichment Analysis (GSEA) (v4.1.0) tool was used for the GSEA enrichment analysis of all genes, and a GSEA enrichment analysis pathway map was drawn.

### 2.5. Real-Time Fluorescence Quantitative PCR (qPCR) Validation

RNA extraction from liver and muscle tissues was performed using the TRIzol Reagent Kit (Thermo Fisher Scientific Inc., Waltham, MA, USA) and then reverse transcribed using a ReverTra Ace M-MLV kit (TOYOBO, Osaka, Japan) according to the manufacturer’s protocol. The primers used are listed in Supplementary Materials Table S1. The qPCR was performed on a Bio-Rad CFX96 Real-Time PCR System (Bio-Rad, Hercules, CA, USA) under the following conditions: 95 °C for 2 min, followed by 40 cycles of 95 °C, 15 s; 60 °C, 20 s; 72 °C, 30 s. Three biological replicates were used for each gene and the expression values relative to *β-actin* were calculated using the 2^−ΔΔCt^ method, as described previously [18].

### 2.6. Statistical Analysis

Experimental data are presented as the mean ± standard error of the mean (SEM). GraphPad Prism 9.0 software (GraphPad Inc., San Diego, CA, USA) was used for the one-way ANOVA, and statistical significance was set at *p* < 0.05. The qPCR data were analyzed and graphed using GraphPad Prism 9.0 software.

## 3. Results

### 3.1. Muscle Composition

There were no significant differences in moisture content among the three varieties of carp muscle (*p* > 0.05). The ash content was significantly elevated in FFRC muscle compared to YC and HC (*p* < 0.05), though YC and HC did not differ significantly from each other (*p* > 0.05). In terms of crude lipid, FFRC muscle exhibited the highest levels, followed by YC, with HC displaying the lowest; nonetheless, these differences did not reach statistical significance across the three varieties of carp (*p* > 0.05). Regarding crude protein, HC muscle registered the highest content, followed by FFRC, and YC had the lowest. There was a significant difference in crude protein content between HC and YC muscles (*p* < 0.05), but no significant differences were observed between FFRC and both HC and YC (*p* > 0.05) (Table 1).

As shown in Table 2, the total amino acid content was highest in HC muscle, followed by FFRC, and lowest in YC, with significant differences in total amino acid content among the three varieties of carp muscle (*p* < 0.05). Similarly, the non-essential amino acid content, umami amino acid content, and sweet amino acid content were significantly elevated in HC muscle compared to both FFRC and YC (*p* < 0.05). The essential amino acid content and bitter amino acid content in HC muscle showed no significant difference compared to FFRC (*p* > 0.05) but were significantly higher than in YC (*p* < 0.05). Specifically, the concentrations of alanine (Ala), arginine (Arg), aspartic acid (Asp), glycine (Gly), lysine (Lys), phenylalanine (Phe), serine (Ser), and tyrosine (Tyr) were significantly elevated in HC muscle compared to both FFRC and YC (*p* < 0.05). The contents of cysteine (Cys), histidine (His), and methionine (Met) in HC muscle showed no significant difference compared to FFRC (*p* > 0.05) but were significantly higher than in YC (*p* < 0.05).

As shown in Table 3, there were no significant differences in the total amounts of saturated fatty acids and monounsaturated fatty acids among the three varieties of carp muscle (*p* > 0.05). Similarly, the concentrations of EPA were consistent across the groups, with no significant variations observed (*p* > 0.05). Regarding DHA, the levels in FFRC and HC muscles did not differ significantly (*p* > 0.05); however, both were significantly elevated compared to YC (*p* < 0.05). Additionally, the concentrations of C18:2n6c, C20:3n6, C20:4n6, C22:5N6 (DPAn-6), C22:5N3 (DPAn-3), total n-3 polyunsaturated fatty acids (n-3ΣPUFA), and total n-6 polyunsaturated fatty acids (n-6ΣPUFA) in HC and YC muscles were not significantly different (*p* > 0.05), yet all were markedly lower than those found in FFRC (*p* < 0.05).

### 3.2. An Overview of the Liver and White Muscle Transcriptome Data

In this study, transcriptome sequencing was performed on nine liver and nine muscle samples, resulting in a total acquisition of 52.49 gigabases (Gb) of clean data. The effective data volume for each sample ranged from 2.52 Gb to 3.42 Gb. The percentage of Q30 exceeded 95.0%. These clean reads were compared to the genome sequence of *Cyprinus carpio*, with a comparison rate exceeding 88.95% (Supplementary Materials Table S2). These results indicate that the data quality obtained from this experiment satisfied further analysis.

**Figure 1:**
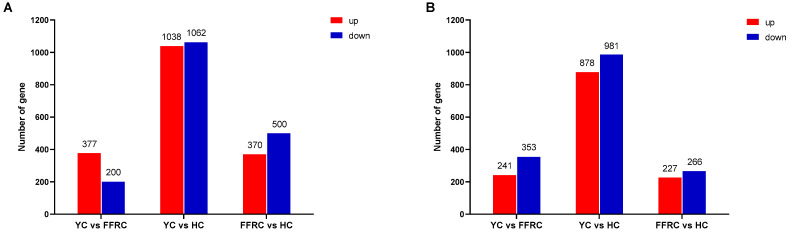
Comparison of differential gene expression in the different carps of liver and muscle samples. Bar graphs (**A**) (liver) and (**B**) (muscle) show different comparisons of upregulated and downregulated genes.

Analysis of differentially expressed genes (DEGs) in the liver tissues of three varieties of carp indicated that compared to YC, FFRC exhibited 577 DEGs, comprising 377 upregulated and 200 downregulated genes. Compared to YC, HC showed 2100 DEGs, including 1038 upregulated and 1062 downregulated genes. When comparing HC to FFRC, there were 870 DEGs, with 370 genes upregulated and 500 genes downregulated (Figure 1A).

Differential gene expression analysis of muscle tissues from three varieties of carp indicated that compared to YC, FFRC had 594 DEGs, of which 241 were upregulated and 353 were downregulated. When comparing HC to YC, there were 1859 DEGs, with 878 genes upregulated and 981 genes downregulated. Between HC and FFRC, there were 493 DEGs, including 227 upregulated and 266 downregulated (Figure 1B).

### 3.3. GO Enrichment Analysis

**Figure 2:**
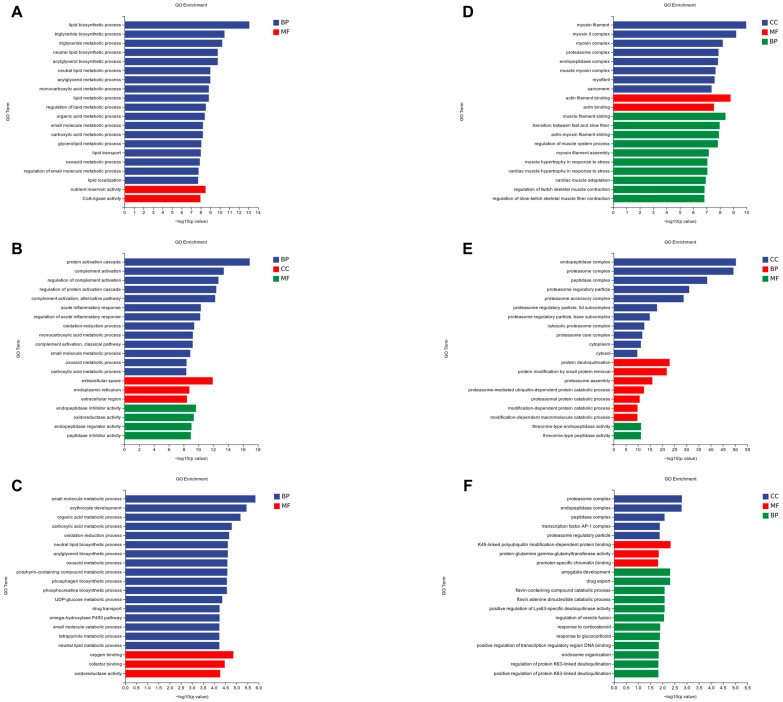
Illustration of the GO pathways in the different carps of liver and muscle samples. (**A**–**C**) represent the results of pairwise comparisons between the liver tissues of groups YC, FFRC, and HC, while (**D**–**F**) represent the results of pairwise comparisons between muscle tissues of groups YC, FFRC, and HC. The horizontal axes in the graphs display the names of the GO entries, and the vertical axes indicate the −log10 *p* values.

GO enrichment analysis of liver tissue showed that DEGs between YC and FFRC were enriched in biological processes related to lipid metabolism (Figure 2A). In the comparison between YC and HC, the DEGs predominantly clustered in pathways involved in protein activation cascade, complement activation, and extracellular space (Figure 2B). For the comparison between FFRC and HC, the genes showed significant enrichment in small molecule metabolic process, erythrocyte development, and oxygen binding (Figure 2C).

In muscle tissues, GO enrichment analysis demonstrated that the genes differentially expressed between YC and FFRC were notably concentrated in myosin filament, actin filament binding, and muscle filament sliding (Figure 2D). Between YC and HC, the enriched categories primarily included endopeptidase complex, proteasome complexes, and protein deubiquitination (Figure 2E). For FFRC versus HC, the analysis revealed significant enrichment in proteasome complexes, endopeptidase complexes, and K48-linked, polyubiquitin modification-dependent protein binding (Figure 2F).

### 3.4. KEGG Enrichment Analysis

**Figure 3:**
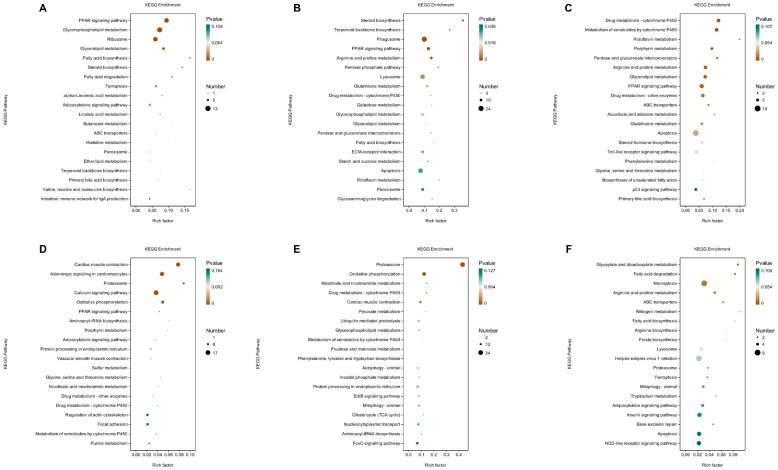
KEGG bubble plots representing the different carps of liver and muscle samples. (**A**–**C**) present the results of two-by-two comparisons between the liver tissues of groups YC, FFRC, and HC, while (**D**–**F**) show the outcomes of similar comparisons between the muscle tissues of groups YC, FFRC, and HC. The horizontal axes in the graphs represent the enrichment scores, where larger bubbles indicate higher numbers of differentially protein-coding genes within that entry. The bubble color transitions from green to white and then to brown with smaller enrichment *p*-values indicate greater statistical significance values.

KEGG pathway enrichment analysis of liver tissues revealed significant enrichment in DEGs between YC and FFRC within the PPAR signaling pathway, as well as various metabolic pathways. These include glycerophospholipid metabolism, fatty acid biosynthesis, alpha-linolenic acid metabolism, histidine metabolism, and valine, leucine, and isoleucine biosynthesis (Figure 3A). In the comparison between YC and HC, the DEGs showed significant enrichment in the phagosome, lysosome, PPAR signaling pathway, and multiple metabolic pathways such as arginine and proline metabolism, glycerophospholipid metabolism, glutathione metabolism, and galactose metabolism (Figure 3B). For FFRC versus HC, the analysis identified enriched pathways including apoptosis, PPAR signaling, and several metabolic pathways, notably glycerolipid metabolism, arginine, and proline metabolism, phenylalanine metabolism, and glycine, serine, and threonine metabolism (Figure 3C).

KEGG pathway enrichment analysis of muscle tissues showed that the DEGs between YC and FFRC were enriched in the calcium signaling pathway, PPAR signaling pathway, muscle contraction-related pathways, and several metabolic pathways, such as oxidative phosphorylation, and glycine, serine, and threonine metabolism (Figure 3D). In the comparison between YC and HC, the DEGs showed significant enrichment in the ErbB signaling pathway, FoxO signaling pathway, and multiple metabolic pathways including oxidative phosphorylation, phenylalanine, tyrosine and tryptophan biosynthesis, and glycerophospholipid metabolism (Figure 3E). For FFRC versus HC, the DEGs were enriched in necrosis and several metabolic pathways such as fatty acid degradation, arginine and proline metabolism, arginine biosynthesis, and tryptophan metabolism (Figure 3F).

### 3.5. DEGs in Fatty Acid Metabolism and Amino Acid Metabolism-Related Pathways

**Figure 4:**
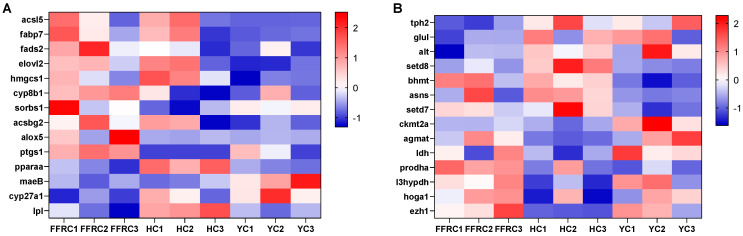
Heatmaps illustrating the changes in gene expression related to (**A**) fatty acid metabolism in the liver and (**B**) amino acid metabolism in the muscle. The FPKM value of each gene was used to plot the heatmaps. Upregulated genes are visually represented in red, whereas downregulated genes are represented in blue.

Heatmaps provide a visual representation of the DEGs associated with the fatty acid metabolism-related pathways in the liver (Figure 4A) and amino acid metabolism-related pathways in the muscle (Figure 4B) across three carp varieties. Analyzing these variations in DEGs will contribute to a deeper understanding of the molecular foundations underlying nutritional deposition in carp muscle tissue.

### 3.6. Verification of the DEGs

**Figure 5:**
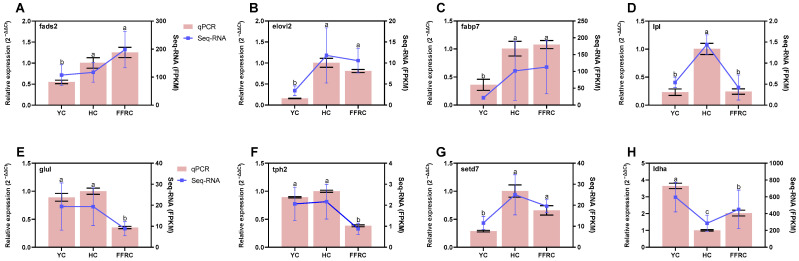
Relative expressions of DEGs detected by qPCR and RNA-Seq. The left y-axes represent the relative expression results obtained via qPCR. The right y-axes show the gene expression levels in FMPK obtained through RNA-Seq. (**A**–**D**) correspond to the four validated genes in the liver tissues while (**E**–**H**) represent the four validated genes in the muscle tissues. Different lowercase letters represent significant differences (*p* < 0.05), and the letters apply only to the comparison of qPCR data.

To validate the gene expression variations observed in the transcriptomic analyses, this study selected four genes associated with fatty acid metabolism in the liver and four genes related to amino acid metabolism in the muscle for qPCR analysis. The purpose of validating the expression of these chosen genes was to verify the accuracy of the transcriptomic data and to enhance the understanding of their regulatory functions in fatty acid metabolism in carp liver and amino acid metabolism in muscle tissue. The expression of *fads2*, *elovl2*, and *fabp7* was upregulated in the FFRC liver, whereas the expression of *lpl* was downregulated (Figure 5A–D). In HC muscle, the expression of *glul*, *tph2*, and *setd7* was upregulated, and the expression of *ldha* was downregulated (Figure 5E–H). The expression patterns of these genes were consistent with the transcriptomic analysis, thereby corroborating our interpretation of the transcriptomic data and supporting subsequent biological inferences.

## 4. Discussion

Fish not only provide macronutrients such as protein but also micronutrients including PUFAs and vitamins, making a significant contribution to the global food and nutrition supply [19]. This research endeavored to identify key factors and signaling pathways associated with nutritional deposition by comparing the muscle composition and transcriptomic profiles of three distinct carp varieties. The findings are intended to furnish a theoretical foundation for genetic enhancements aimed at optimizing the nutritional quality of carp.

### 4.1. Fatty Acid Metabolism

Omega-3 PUFAs, such as EPA and DHA, have been extensively reported to exert beneficial effects on cardiovascular health [20] and to mitigate the risks associated with inflammation and chronic diseases [21]. Similarly, omega-6 PUFAs, although potentially pro-inflammatory when consumed in excess, are crucial for health when maintained in appropriate ratios to omega-3 PUFAs [22]. This study demonstrated that FFRC muscle exhibited the highest concentrations of n-3ΣPUFA and n-6ΣPUFA, suggesting that FFRC is capable of efficiently synthesizing these crucial fatty acids. Consequently, FFRC offers a superior source of omega-3 and omega-6 PUFAs, which is significantly beneficial for consumers in search of nutritious dietary options.

Fatty acid metabolism in carp involves a complex network of multiple metabolic pathways and transcription factors. Transcriptomic analysis indicated a significant upregulation of genes associated with fatty acid metabolism in the liver of FFRC. The peroxisome proliferator-activated receptor (PPAR) signaling pathway is integral to the regulation of lipid metabolism. PPARs modulate the synthesis and metabolism of fatty acids through the transcriptional regulation of pertinent genes [23]. ELOVL fatty acid elongase 2 (ELOVL2) and fatty acid desaturase 2 (FADS2) are enzymes critical to the biosynthetic pathways of long-chain PUFAs. ELOVL2 catalyzes the elongation of fatty acids, while FADS2 facilitates the conversion of saturated fatty acids into unsaturated forms. These enzymes are intimately linked with the production of ω-3 and ω-6 PUFAs [24,25]. Notably, the significant upregulation of these two genes in the liver of FFRC indicates its potential capacity for synthesizing unsaturated fatty acids. Additionally, FADS2 and ELOVL2 are pivotal in the synthesis of unsaturated fatty acids in species like Atlantic cod (*Gadus morhua*) and Atlantic salmon (*Salmo salar*), which are known for their high content of unsaturated fatty acids [26,27]. This highlights the universal significance of these enzymes in fish lipid metabolism.

Furthermore, fatty acid-binding proteins (FABPs) serve a critical function in the transport and signaling of fatty acids [28]. The increased expression of *fabp7* indicated an enhanced intracellular capability for transporting unsaturated fatty acids, facilitating their efficient metabolism and storage. This enhanced capacity may have contributed to the elevated PUFA content observed in the muscle tissues. Acyl-CoA synthetase long-chain family member 5 (ACSL5) and acyl-CoA synthetase bubblegum family member 2 (ACSBG2) are enzymes that activate long-chain fatty acids to their CoA derivatives, promoting their incorporation into metabolic pathways [29,30]. The upregulation of these genes suggests improved activation and utilization of long-chain fatty acids in the metabolic processes of the FFRC liver, resulting in the increased accumulation of unsaturated fatty acids in muscle tissues. Additionally, hydroxymethylglutaryl-CoA synthase 1 (HMGCS1) and sterol 12-alpha-hydroxylase (CYP8B1), which participate in cholesterol metabolism [31,32], and their upregulated expression, may play a critical role in lipid homeostasis. The elevated expression of these genes in the FFRC liver indicated an active mechanism for fatty acid synthesis and regulatory processes.

PPARα is pivotal in the regulation of fatty acid β-oxidation, promoting the catabolism of fatty acids to generate energy [33]. The downregulation of *pparα* in the FFRC liver was likely aimed at augmenting the reserves of unsaturated fatty acids. Lipoprotein lipase (LPL) enzymatically hydrolyzes triglycerides present in lipoproteins to release free fatty acids [34]. Additionally, sterol 26-hydroxylase (CYP27A1) participates in cholesterol metabolism [35]. The downregulation of these genes may have been a regulatory strategy intended to preserve the overall dynamic equilibrium of lipids within the organism.

Overall, these alterations in gene expression led to enhanced deposition of omega-3 and omega-6 PUFAs in FFRC muscle. The upregulation of genes involved in elongation and desaturation processes is critical for PUFA synthesis. Furthermore, the increased expression of genes related to fatty acid transport and activation ensures an ample supply of raw materials for PUFA biosynthesis. Concurrently, the downregulation of genes involved in fatty acid catabolism indicates a shift towards fatty acid storage rather than utilization.

### 4.2. Amino Acid Metabolism

Fish constitute a crucial source of proteins and amino acids within the human diet [19], providing essential nutrients that contribute to muscle quality improvement, immune system enhancement, and cardiovascular health [36,37]. Furthermore, the concentration of essential amino acids serves as a critical nutritional indicator in fish muscle [38]. This study revealed that the levels of crude protein, total amino acids, and essential amino acids in HC muscle were significantly elevated compared to those in FFRC and YC, indicating that HC may possess substantial potential as a high-quality source of protein. Moreover, the flavor profile of fish is intricately linked to the types and concentrations of amino acids, particularly those that enhance flavor [39]. This study found that the levels of umami and sweet amino acids in HC were significantly higher than in FFRC and YC, suggesting that HC could provide enhanced umami and sweet flavors during culinary preparation.

At the metabolic level, muscle transcriptomic analyses revealed a significant upregulation of genes associated with amino acid metabolism in HC. Asp is a critical contributor to the umami flavor, enhancing the palatability of fish muscle [40]. Asparagine synthetase (ASNS) catalyzes the synthesis of asparagine from Asp [41]. The upregulation of *asns* in HC indicated an augmented capacity for asparagine synthesis. Asparagine can be converted to aspartate, which is crucial for protein synthesis and the interconversion of amino acids [42]. This may have explained the elevated levels of Asp observed in HC. Additionally, Ala is known to contribute to the sweet taste [43]. Alanine aminotransferase (ALT) catalyzes the transamination of alanine to pyruvate, which is a reversible reaction [44]. Concurrently, lactate dehydrogenase A (LDHA) catalyzes the conversion of pyruvate to lactate in anaerobic glycolysis, with pyruvate also serving as a substrate for Ala synthesis [45,46]. The upregulation of *alt* and the downregulation of *ldha* in HC may be closely associated with an increase in muscle Ala content.

Furthermore, glutamine synthetase (GS, encoded by *glul*) catalyzes the formation of glutamine from glutamate and ammonia [47]. The upregulation of *glul* in HC indicated enhanced glutamine production, which is essential for effective nitrogen metabolism, muscle growth, and repair. Histone-lysine N-methyltransferase (HMT, encoded by *setd*) and betaine-homocysteine methyltransferase (BHMT) participate in the methylation of amino acids, influencing the expression of pertinent genes [48,49]. The upregulation of these genes related to methylation may have led to alterations in the expression of amino acid metabolism genes in HC muscle, consequently impacting amino acid deposition and protein biosynthesis. Proline dehydrogenase (PRODH), trans-L-3-hydroxyproline dehydratase (L3HYPDH), and 4-hydroxy-2-oxoglutarate aldolase 1 (HOGA1) play roles in the catabolism of proline and hydroxyproline, which are key for collagen synthesis [50,51,52]. The downregulation of these genes suggested an increase in collagen content within HC muscle, not only promoting muscle growth but potentially also elevating the levels of Gly derived from collagen.

The upregulation and downregulation of these key genes suggest a coordinated regulation of metabolic pathways that enhance the deposition of amino acids in HC muscle. The upregulated genes involved in amino acid synthesis and metabolism ensure a steady supply of essential and non-essential amino acids, supporting increased protein synthesis and muscle growth. On the other hand, the downregulated genes indicate shifts in metabolic processes, favoring pathways that enhance amino acid availability for muscle development rather than their catabolism or alternative uses.

In conclusion, the differential expressions of key genes involved in fatty acid and amino acid metabolism provide valuable insights for molecular breeding programs aimed at enhancing the nutritional quality of the common carp. The identified genes, such as *elovl2*, *fads2*, *fabp7*, *pparα*, and *cyp27a1* for PUFAs, and *asns*, *alt*, *glul*, *prodh*, and *hoga1* for amino acids, can serve as potential genetic markers for selecting and breeding strains with superior profiles. Using advanced genetic and genomic tools, such as CRISPR/Cas9 gene editing and marker-assisted selection, it is possible to develop new strains of the common carp with optimized fatty acid and amino acid compositions, thereby enhancing their market value and health benefits.

## 5. Conclusions

This study conducted a comprehensive analysis of the muscle nutritional composition and transcriptome data across three different carp varieties, providing profound insights into the critical genes regulating carp muscle nutritional deposition. The findings revealed that the elevated levels of n-3ΣPUFA and n-6ΣPUFA in FFRC muscle were closely linked with enhanced capabilities in the activation and transportation of fatty acid (*fabp7*, *acsl5*, *acsbg2*) as well as biosynthesis and elongation of long-chain unsaturated fatty acids (*elovl2*, *fads2*) within the liver. The HC variety exhibited significantly higher concentrations of amino acids and crude protein in its muscle compared to the other varieties. Transcriptomic analyses indicated significant alterations in the expression of genes associated with amino acid metabolism (*asns*, *alt*, *ldha*, *glul*, *setd*, *prodh*, *l3hypdh*, *hoga1*) in HC muscle. Overall, these findings enrich our understanding of fatty acid and amino acid biosynthesis and accumulation in carp. Future studies could further investigate the functional roles and regulatory mechanisms of these genes associated with fatty acid and amino acid metabolism, thereby providing new molecular breeding strategies for the genetic enhancement of high-quality carp.

## Figures and Tables

**Table 1:** White muscle chemical composition.

	YC	HC	FFRC
Crude protein (%)	13.09 ± 1.41 ^b^	16.24 ± 0.29 ^a^	14.72 ± 1.40 ^ab^
Crude lipid (%)	3.87 ± 0.43	3.59 ± 1.02	4.64 ± 1.37
Ash (%)	10.58 ± 0.16 ^b^	10.36 ± 0.03 ^b^	11.07 ± 0.19 ^a^
Moisture (%)	77.60 ± 0.19	78.27 ± 0.73	78.56 ± 0.72

Lowercase letters in the same row indicate significant differences between different varieties (*p* < 0.05).

**Table 2:** White muscle amino acid composition (ng/mg wet matter).

Free Amino Acid	YC	HC	FFRC
Alanine	108.43 ± 11.52 ^c^	194.61 ± 14.47 ^a^	162.40 ± 10.92 ^b^
Arginine	9.61 ± 1.45 ^c^	24.32 ± 3.20 ^a^	14.42 ± 3.07 ^b^
Aspartic acid	5.99 ± 0.92 ^c^	30.63 ± 2.35 ^a^	24.72 ± 4.30 ^b^
Cysteine	0.12 ± 0.02 ^b^	0.37 ± 0.19 ^a^	0.30 ± 0.13 ^a^
Glutamic acid	52.95 ± 6.31 ^a^	60.69 ± 6.20 ^a^	43.37 ± 3.58 ^b^
Glycine	289.69 ± 17.01 ^c^	567.65 ± 34.23 ^a^	455.18 ± 47.44 ^b^
Histidine	730.40 ± 106.65 ^b^	840.01 ± 137.40 ^a^	822.00 ± 112.98 ^a^
Isoleucine	11.50 ± 3.26	14.73 ± 2.67	12.01 ± 1.76
Leucine	28.94 ± 2.97 ^b^	37.79 ± 2.34 ^a^	32.37 ± 1.72 ^ab^
Lysine	68.68 ± 1.17 ^c^	150.29 ± 8.02 ^a^	113.30 ± 12.79 ^b^
Methionine	2.16 ± 0.66 ^b^	5.77 ± 0.79 ^a^	4.70 ± 0.46 ^a^
Phenylalanine	6.10 ± 0.59 ^b^	12.23 ± 0.95 ^a^	7.47 ± 1.03 ^b^
Proline	44.81 ± 3.82	38.18 ± 6.47	47.96 ± 9.32
Serine	23.08 ± 2.09 ^c^	48.66 ±6.14 ^a^	35.25 ± 3.55 ^b^
Threonine	54.59 ± 3.99 ^b^	61.87 ± 2.36 ^ab^	69.77 ± 4.93 ^a^
Tyrosine	4.02 ± 0.50 ^c^	10.59 ± 0.74 ^a^	5.94 ± 0.58 ^b^
Valine	18.58 ± 2.05	21.49 ± 2.30	19.69 ± 3.29
^1^∑EAA	930.68 ± 38.87 ^b^	1168.49 ± 28.80 ^a^	1095.74 ± 32.18 ^a^
^2^∑NEAA	529.07 ± 35.79 ^c^	951.38 ± 36.20 ^a^	775.13 ± 28.70 ^b^
^3^∑Umami AA	457.05 ± 23.08 ^c^	853.58 ± 23.41 ^a^	685.67 ± 15.16 ^b^
^4^∑Sweet AA	589.27 ± 33.09 ^c^	1061.26 ± 39.50 ^a^	883.88 ± 16.20 ^b^
^5^∑Bitter AA	824.90 ± 25.29 ^b^	991.27 ± 32.19 ^a^	939.45 ± 34.18 ^a^
^6^TAA	1459.75 ± 42.48 ^c^	2119.87 ± 48.19 ^a^	1870.87 ± 48.79 ^b^

Lowercase letters in the same row indicate significant differences between different varieties (*p*  <  0.05). ^1^∑EAA: the sum of essential amino acids (Lys, Met, Thr, Arg, Leu, His, Ile, Phe, and Val); ^2^∑NEAA: the sum of non-essential amino acids (Asp, Ser, Glu, Ala, Cys, Gly, Tyr, and Pro); ^3^∑Umami AA: the sum of umami amino acids (Asp, Glu, Gly, and Ala); ^4^∑Sweet AA: the sum of sweet amino acids (Gly, Ala, Ser, Thr, Lys, and Pro); ^5^∑Bitter AA: the sum of bitter amino acids (Met, Val, Leu, Ile, Phe, Ser, Tyr, and His); ^6^TAA: total amino acids.

**Table 3:** White muscle fatty acid composition (ng/mg wet matter).

Fatty Acid	YC	HC	FFRC
C14:0	12.49 ± 4.91	5.65 ± 0.62	8.84 ± 5.14
C15:0	6.45 ± 0.5	6.41 ± 0.52	9.27 ± 3.38
C16:0	171.17 ± 13.74	164.02 ± 8.07	191.66 ± 29.74
C17:0	4.6 ± 0.34 ^b^	5.86 ± 1.00 ^ab^	7.03 ± 1.59 ^a^
C18:0	70.96 ± 6.27	79.95 ± 7.95	81.4 ± 24.96
C20:0	1.32 ± 0.17	1.81 ± 0.81	1.76 ± 0.93
C22:0	0.16 ± 0.03	0.15 ± 0.05	0.12 ± 0.02
Total saturated fatty acids	267.13 ± 22.39	263.85 ± 15.57	300.09 ± 33.22
C16:1	19.34 ± 5.48	13.2 ± 4.82	16.46 ± 10.99
C18:1n9c	211.47 ± 53.28	154.57 ± 18.00	192.11 ± 52.21
C22:1n9	4.3 ± 1.68	7.52 ± 4.1	3.99 ± 1.42
Total monounsaturated fatty acids	235.11 ± 60.42	175.3 ± 24.84	212.56 ± 63.43
C18:3n6	4.16 ± 0.32	2.66 ± 0.75	4.37 ± 1.13
C18:3n3	46.51 ± 14.44	29.4 ± 3.07	44.09 ± 7.48
C18:2n6c	169.06 ± 11.43 ^b^	150.63 ± 9.81 ^b^	196.3 ± 17.23 ^a^
C20:2	16.48 ± 1.12	17.66 ± 1.76	20.76 ± 3.46
C20:3n6	75.83 ± 4.49 ^b^	73.73 ± 10.79 ^b^	90.62 ± 11.2 ^a^
C20:4n6	90.82 ± 3.47 ^b^	89.44 ± 4.07 ^b^	113.13 ± 7.44 ^a^
C22:4n6	6.49 ± 0.99	11.20 ± 1.25	12.07 ± 1.84
C22:5N6(DPAn-6)	28.11 ± 1.86 ^b^	29.99 ± 3.85 ^b^	37.8 ± 2.33 ^a^
C22:5N3(DPAn-3)	11.55 ± 1.04 ^b^	14.45 ± 1.71 ^b^	18.25 ± 1.20 ^a^
EPA	15.11 ± 1.09	15.84 ± 4.95	17.55 ± 4.13
DHA	89.93 ± 2.04 ^b^	117.69 ± 4.17 ^a^	119.00 ± 8.58 ^a^
Total polyunsaturated fatty acids	554.05 ± 26.44 ^b^	552.7 ± 27.12 ^b^	673.93 ± 33.97 ^a^
DHA + EPA	105.04 ± 3.09 ^b^	133.53 ± 3.42 ^a^	136.55 ± 9.75 ^a^
n-3ΣPUFA	163.1 ± 9.87 ^b^	177.39 ± 6.77 ^b^	198.89 ± 9.55 ^a^
n-6ΣPUFA	374.48 ± 9.39 ^b^	357.65 ± 22.60 ^b^	453.95 ± 26.98 ^a^

Lowercase letters in the same row indicate significant differences between different varieties (*p* < 0.05).

## Data Availability

The raw sequence data reported in this paper were deposited in the Genome Sequence Archive in the National Genomics Data Center, China National Center for Bioinformation/Beijing Institute of Genomics, Chinese Academy of Sciences (GSA: CRA016317) and are publicly accessible at (https://ngdc.cncb.ac.cn/gsa) (accessed on 8 May 2020).

## Supplementary Materials

The following supporting information can be downloaded at: https://www.mdpi.com/article/10.3390/ani14131939/s1, Table S1: qPCR primer sequences; Table S2: Results of the quality data sequencing.
